# New Appliances and Things Medical

**Published:** 1898-03-12

**Authors:** 


### NEW APPLIANCES AND THINGS MEDICAL.

[We shall be glad to receive, at our Office, 28 & 29, Southampton Street, Strand, London, W.C., from the manufacturers, specimens of all new preparations and appliances which may be brought out from time to time.]

### MARVINE GELOIDS.

(Marvine, Limited, 28, Eldon Street, E.C.)

Marvine geloids are in the form of a lozenge which readily dissolves in the mouth and have a very pleasant acid flavour. They are said to be nutritious, stimulating, thirst quenching, and delectable. On examination they gave decided evidence of containing meat extractives and cocoa in a very palatable form. As a lozenge for allaying thirst, at the same time being of some nutritive value, Marvine geloids should be appreciated by cyclists and travellers generally, and in some cases even by invalids, as being more serviceable than the usual kind of meat lozenge.

### VITÆ ORE.

The Vitæ Ore Company, 39, Temple Chambers, Temple Avenue, E C.)

Vitæ ore is a mineral from the United States, which is suggested as applicable for medicinal purposes. It consists of a siliceous ore containing iron sulphates which have been produced by a natural course of oxidation from the originally existing iron sulphide. The analysis of the ore shows it to consist of about 18½ per cent. of constituents soluble in water containing 16⅔ per cent. iron salts and 0·75 per cent. sulphates of magnesia and soda. The therapeutic value of the ore is solely in the portion which is dissolved by water, and it is directed that the contents of one bottle, about one ounce of the ore, be added to a quart of water, and the "elixir," that is the aqueous extract, be taken in doses of one teaspoonful to one tablespoonful in half a tumbler of boiling water. A sample of the elixir submitted to us was an acid solution containing 3 3 grains of solids per fluid ounce, of which 2½ grains were iron sulphates. There is no doubt that where iron salts are indicated for medicinal purposes the elixir of vitæ ore would serve the same purpose as the ferrous and ferric sulphates of the Pharmacopœia, but whether reverting to the primitive means suggested by the use of this material will be acceptable in these days when elegant pharmacy is a desideratum is a matter of individual opinion. One ounce of vitæ ore, sufficient to make one quart of the elixir, costs 4s. 6d.

